# *Btbd3* expression regulates compulsive-like and exploratory behaviors in mice

**DOI:** 10.1038/s41398-019-0558-7

**Published:** 2019-09-09

**Authors:** Summer L. Thompson, Amanda C. Welch, Emily V. Ho, João M. Bessa, Carlos Portugal-Nunes, Mónica Morais, Jared W. Young, James A. Knowles, Stephanie C. Dulawa

**Affiliations:** 1 Department of Psychiatry, University of California San Diego, La Jolla, CA 92093 USA; 2 Committee on Neurobiology, The University of Chicago, Chicago, IL 60637 USA; 3 Life and Health Sciences Research Institute (ICVS), School of Medicine, University of Minho, Braga, Portugal; 4 ICVS/3B’s - PT Government Associate Laboratory, Braga, Guimarães Portugal; 5 Department of Cell Biology, SUNY Downstate Medical Center College of Medicine, Brooklyn, NY 11203 USA

**Keywords:** Psychiatric disorders, Neuroscience

## Abstract

BTB/POZ domain-containing 3 (*BTBD3*) was identified as a potential risk gene in the first genome-wide association study of obsessive-compulsive disorder (OCD). BTBD3 is a putative transcription factor implicated in dendritic pruning in developing primary sensory cortices. We assessed whether BTBD3 also regulates neural circuit formation within limbic cortico-striato-thalamo-cortical circuits and behaviors related to OCD in mice. Behavioral phenotypes associated with OCD that are measurable in animals include compulsive-like behaviors and reduced exploration. We tested *Btbd3* wild-type, heterozygous, and knockout mice for compulsive-like behaviors including cage-mate barbering, excessive wheel-running, repetitive locomotor patterns, and reduced goal-directed behavior in the probabilistic learning task (PLT), and for exploratory behavior in the open field, digging, and marble-burying tests. *Btbd3* heterozygous and knockout mice showed excessive barbering, wheel-running, impaired goal-directed behavior in the PLT, and reduced exploration. Further, chronic treatment with fluoxetine, but not desipramine, reduced barbering in *Btbd3* wild-type and heterozygous, but not knockout mice. In contrast, *Btbd3* expression did not alter anxiety-like, depression-like, or sensorimotor behaviors. We also quantified dendritic morphology within anterior cingulate cortex, mediodorsal thalamus, and hippocampus, regions of high *Btbd3* expression. Surprisingly, *Btbd3* knockout mice only showed modest increases in spine density in the anterior cingulate, while dendritic morphology was unaltered elsewhere. Finally, we virally knocked down *Btbd3* expression in whole, or just dorsal, hippocampus during neonatal development and assessed behavior during adulthood. Whole, but not dorsal, hippocampal *Btbd3* knockdown recapitulated *Btbd3* knockout phenotypes. Our findings reveal that hippocampal *Btbd3* expression selectively modulates compulsive-like and exploratory behavior.

## Introduction

Obsessive-compulsive disorder (OCD) is a psychiatric disorder characterized by intrusive and unwanted thoughts, images, or urges and/or compulsive behaviors^1^. Although OCD is often comorbid with anxiety disorders^2^, the fifth edition of the *Diagnostic and Statistical Manual of Mental Disorders* (DSM-5) reclassified OCD into a new category of disorders in which compulsive behavior is the core feature, consistent with recent hypotheses that compulsivity may comprise a transdiagnostic psychiatric trait^3,4^. Compulsive behaviors are defined as maladaptive repetitive behaviors that are performed despite no longer leading to a goal, and are typically inflexible in nature^5–7^. Unlike obsessions, compulsive behavior can be operationalized in rodent models. For one, chronic treatment with serotonin reuptake inhibitors (SRIs), but not other classes of antidepressants including norepinephrine reuptake inhibitors (NRIs), provide effective treatment for OCD^8^, and reduce compulsive-like behaviors in rodents including route stereotypy, repetitive jumping, and flipping^9–12^. Furthermore, aspects of compulsive behavior can be measured directly in rodents, such as neuropsychological endophenotypes in cognitive tasks. Our knowledge of the neurobiological underpinnings of compulsive behavior remain incomplete, and animal models provide an important tool for revealing these mechanisms.

The first genome-wide association study (GWAS) of OCD identified rs6131295, a single nucleotide polymorphism (SNP) located near BTB/POZ domain-containing 3 (*BTBD3*), as genome-wide significant in the trio, but not the case-control, portion of the study^13^. This SNP was also an expression quantitative trait locus (eQTL) for *BTBD3*^13^, but was not significant in a second GWAS^14^ or a meta-analysis^15^. However, these GWASs were perhaps insufficiently powered to identify genome-wide significant loci. The function of BTBD3 has been largely unknown, until a recent report identified a role for BTBD3 in neurodevelopment. BTBD3 was found to orient dendrites toward active axon terminals and regulate activity-dependent dendritic pruning in primary sensory cortex during neonatal development, thus facilitating neural circuit formation^16^. Yet, whether BTBD3 also performs this function in other brain regions in which it is highly expressed, including limbic cortico-striato-thalamo-cortical (CSTC) circuits, remains unknown.

In humans and rodents, *BTBD3* is highly expressed within limbic CSTC circuitry including the mediodorsal thalamus, anterior cingulate cortex (ACC), and hippocampus^17,18^. Within this circuit, *BTBD3* expression increases rapidly during early postnatal development and peaks during childhood and adolescence in humans^18^ and mice^16,17^. Dysfunction within limbic CSTC circuitry is associated with abnormal repetitive behaviors^19,20^, and specifically with compulsive behaviors^21,22^. Furthermore, the hippocampus, mediodorsal thalamus, and ACC are key structures for goal-directed decision-making^23–27^, which is impaired in OCD^28–30^, and theorized to underlie expression of compulsive behaviors^31,32^. Thus, we aimed to determine the role of BTBD3 in the morphological development of this circuit, and in the expression of compulsive-like behaviors.

Herein, we sought to address three key questions: (i) Does *Btbd3* expression modulate behaviors related to OCD, such as compulsive-like behaviors? (ii) Does altering *Btbd3* expression affect neuronal morphology within the ACC, mediodorsal thalamus, or hippocampus? (iii) In which region of the limbic CSTC circuit does *Btbd3* expression regulate behavioral phenotypes observed? To address these questions, we used *Btbd3* wild-type (WT), heterozygous (HT), and knockout (KO) mice to assess behavioral phenotypes and dendritic morphology within the mediodorsal thalamus, ACC, CA1, and dentate gyrus. Finally, we generated and used *Btbd3*^flox^ mice and an adeno-associated virus (AAV) expressing Cre recombinase (Cre) to knockdown (KD) either dorsal or whole hippocampal *Btbd3* expression during the early neonatal period, and assessed behavioral consequences during adulthood.

## Materials and methods

### Animals

Adult (minimum 7 weeks) male and female mice were group-housed by sex on a 12:12 h light:dark cycle (~500 lx overhead lighting in housing room) with ad libitum standard chow and water except during cognitive testing (see below). All testing occurred during the light cycle unless otherwise specified. *Btbd3* KO mice were on a 129/B6 background (RIKEN, Saitama, Japan). Experimental cohorts were bred in-house and generated from littermates from heterozygous (HT) *Btbd3* crosses, resulting in variable group sizes based on breeding production. Minimum sample sizes were targeted based on previous experience with these paradigms. Large cohort sizes were used for assessing barbering, based on previous reports of low barbering frequency in control animals^33^. *Btbd3*-floxed (*Btbd3*^flox^) mice were generated on a pure C57BL/6J background (Transviragen, Research Triangle Park, NC) using CRISPR/Cas9 to insert loxP sites flanking exon 2 of *Btbd3*. Mice weighed 16–35 g. Genotype or viral condition, sex, and home cage were counterbalanced across testing. Sample sizes for each test are listed in the figure legends. All procedures were performed in accordance with the local Institutional Animal Care and Use Committee and the National Institutes of Health Guidelines for the Care and Use of Laboratory Animals.

### Drugs

Drugs were administered in the drinking water in opaque bottles for 14 weeks. The SRI fluoxetine was administered at 80 mg/L to achieve a 10 mg/kg/day dose and changed weekly. The NRI desipramine was administered at 215 mg/L to achieve a 20 mg/kg/day dose and changed biweekly^34,35^. Fluoxetine and desipramine were used because chronic treatment with SRIs, but not other classes of antidepressants, serve as effective monotherapy for OCD^36,37^.

### Behavioral studies

#### Barbering

Mice were pair-housed by sex and genotype, photographed weekly, and inspected for evidence of barbering, an abnormal behavior in which animals repetitively clip or pluck the fur of their cage mates, generating bald spots^38^. See Supplementary Information.

#### Cognitive testing

Male mice were food-restricted to 85% of free-feeding bodyweight to motivate responses for food reward, trained to respond on a fixed-ratio 1 (FR1) schedule, and then assessed in a Go/No-Go task to measure response inhibition^39^, progressive ratio breakpoint (PRBP) task to measure motivation for reward^40^, and finally the probabilistic learning task (PLT) to measure goal-directed and habitual decision-making strategies^41^. See Supplementary Information.

#### Wheel-running

In order to track home-cage locomotor activity, mice were singly housed in standard cages equipped with wireless running wheels (Med Associates, St. Albans, VT). Wheel revolution counts were continuously transmitted to a computer running Wheel Manager Software (Med Associates) for 7 days in the mouse housing room. Mice that never acclimated to running wheels (such as burying the wheel in bedding; quantified as fewer than 300 total wheel revolutions for the entire week) were excluded (*n* = 6).

#### Open field

In order to track locomotor activity in a novel environment, mice were placed in a corner of an open field (OF) apparatus (Accuscan, Columbus, OH) equipped with a central overhead light (~100 lx) and activity was monitored for 45 min in 5-min bins. Versamax software (Accuscan, Columbus, OH) generated primary outcome measures including distance traveled (locomotion), vertical rearing (exploration), and center activity (anxiety). The spatial scaling exponent *d* (spatial *d*) was calculated using NightOwl software (Custom) and Python (Python Software Foundation, Beaverton, OR). Spatial *d* describes the smoothness of the path of the animal, where high values indicate many directional changes, whereas low values indicate a more straight and rigid path, characteristic of compulsive circling in the OF^10,11,42^.

#### Dig test

To measure exploration^43^, mice were placed in novel, clean standard cages with fresh bedding (1″ deep) and recorded for 3 min^35^. Videos were scored by a blind observer for digging behavior, defined by significant displacement of bedding using forelimbs.

#### *Marble-burying*

Marble burying was assessed as an additional measure of exploratory digging^44–47^. Fresh cages were filled with 5 cm bedding with 12 marbles placed on top in a 3 × 4 grid with 4 cm center-to-center spacing. Number of marbles buried to 2/3 depth was recorded after 30 min^44^.

#### Nest-building

Nest-building is a measure of well-being in mice^48^. Mice were given a pre-weighed compressed cotton nestlet (Ancare, Bellmore, NY) while singly housed in the home cage^49^. In the 8-h version, nestlets were removed 8 h later and any unused nestlet was weighed. In the overnight version, nestlets were provided just before initiation of the dark cycle. Fourteen hours later, any unused nestlet was weighed.

#### Light-dark test

The light-dark test was performed to assess anxiety-like behavior^50^. The light-dark test was performed in the OF (overhead light adjusted to ~40 lx) using dark inserts to cover half the chamber (Omnitech Electronics, Inc., Columbus, OH, USA). Animals began in the dark side of the chamber and activity was recorded for 10 min. Outcome measures were duration on each side, proportion of distance traveled in the dark, and latency to enter the light side.

#### Novelty-induced hypophagia (NIH)

NIH testing assesses anxiety, and was performed as previously described^51^. Briefly, mice underwent three days of training to consume sweetened condensed milk. The following day, mice were presented with sweetened condensed milk in the home cage for 30 min. Latency to drink and consumption volume were measured. The following day, mice were presented with sweetened condensed milk in a novel cage with no bedding and bright lighting for 30 min. Latency to drink and consumption volume were recorded.

#### Forced swim test (FST)

Animals were tested in the FST to assess depression-like behavior^52^. Mice were placed in opaque plastic buckets (18 cm × 20.5 cm) filled with 23–25 ^o^C water for 6 min. The day prior to testing, animals underwent a 15 min pretest under the same conditions. Each session was recorded. The video from the test day was hand-scored by a blind observer for time spent immobile, swimming, or climbing in the final 4 min.

### Viral-mediated *Btbd3* knockdown

In order to assess the role of hippocampal *Btbd3* expression in behavior, postnatal day 2 *Btbd3*^flox^ mouse pups received intracranial infusions of AAV expressing Cre: AAV2/8-CMV-Cre-P2A-tdTomato-WPRE (AAV-Cre; Viral Vector Core Facility, University of Iowa, Iowa City, IA) or control: AAV2/8-CMV-tdTomato-WPRE (AAV-tdTomato), targeting the whole hippocampus (0.3 mm anterior, 2.0 mm lateral, 2.5 mm ventral of lambda), or only the dorsal half of the hippocampus (1.0 mm anterior, 0.3 mm lateral, 2.7 mm ventral of lambda). See Supplementary Information. Mice were assessed for behavior during adulthood (8–13 weeks) in a subset of paradigms tested in the global *Btbd3* KO mice.

### Statistical analysis

Dependent measures were analyzed using repeated measures or factorial analysis of variance (ANOVA). Significant interactions were resolved by assessing simple main effects for factors with more than two groups with Bonferroni correction. Post hoc tests for between-subjects factors were performed using Student–Newman–Keuls. Where appropriate, Kruskal–Wallis one-way ANOVAs were performed, and Mann–Whitney *U* tests with Bonferroni correction were used as post hoc tests. Categorical dependent measures were analyzed using chi-square tests for endpoint factors which were confirmed with a bootstrap analysis, and Kaplan–Meier survival curves with Mantel–Cox logrank tests for repeated measures analyses. Alpha was set at 0.05. A few mice were excluded from analyses because of experimental errors, but no statistical outliers were removed.

## Results

### *Btbd3* KO mice exhibit compulsive-like behavior

**Fig. 1:**
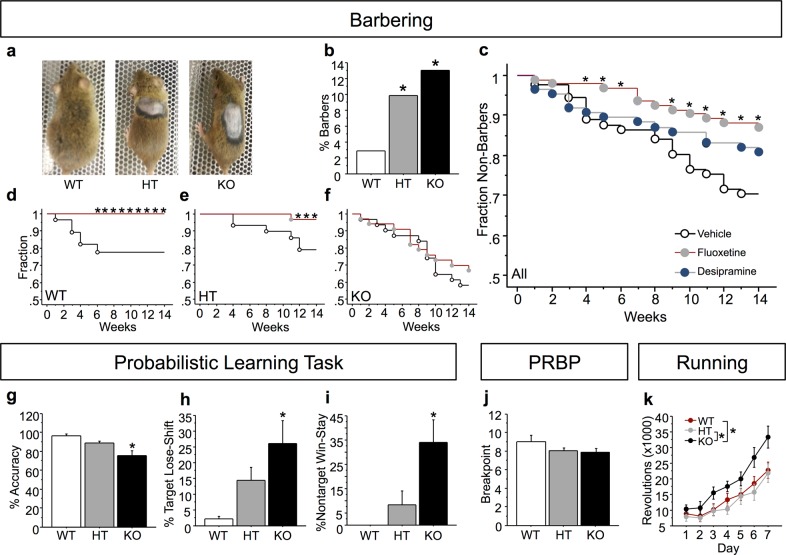
*Btbd3* knockout induces compulsive-like behavior. **a** Representative example photographs of dorsal surface of the coat in BTBD3 WT (left), HT (middle), and KO (right) mice. **b** Increased incidence of barbering behavior was identified in *Btbd3* HT and KO mice in a chi-square analysis (*n* = 81 female/90 male WT, 150 female/165 male HT, 83 female/78 male KO mice). Across genotypes **c**, chronic fluoxetine reduced onset of barbering over time in a cohort of initially 100% non-barbers, whereas desipramine did not significantly alter barbering behavior at any time point in a Kaplan–Meier survival curve analysis (*n* = 15 female/13 male WT, 16 female/15 male HT, and 19 female/12 male KO for vehicle treatment, 19 female/13 male WT, 13 female/12 male HT, and 14 female/16 male KO for desipramine treatment, and 14 female/14 male WT, 16 female/17 male HT, and 19 female/15 male KO mice in the fluoxetine treatment group). Drops indicate time points when mice began to barber a cage mate, with the cumulative fraction of non-barbers indicated on the Y-axis. Within *Btbd3* WT mice **d**, fluoxetine completely prevented onset of barbering behavior. Within *Btbd3* HT mice **e**, fluoxetine-reduced barbering behavior. Within *Btbd3* KO mice **f**, barbering was unaffected by fluoxetine treatment. **g** *Btbd3* KO mice showed reduced accuracy on the PLT (*n* = 13 WT, 16 HT, 17 KO mice, all male), which was driven by an increased frequency of lose-shift responses on the target port **h** and an increased frequency of win-stay responses on the non-target port **i**. **j** Breakpoint was unaffected by Btbd3 genotype in the PRBP task in an ANOVA. **k** *Btbd3* KO mice exhibited excessive wheel-running behavior during the dark cycle (*n* = 10 WT, 9 HT, and 10 KO mice of each sex). Results are expressed as mean values ± SEM, except for panels depicting categorical data, which does not have error. *p < .05 vs. WT or vehicle groups in a chi-square analysis **b**, log-rank analysis **c**–**f**, or an ANOVA with post hoc tests **g**–**k**. WT wild-type, HT heterozygous, KO knockout, PLT probabilistic learning task, PRBP progressive ratio breakpoint task

We observed a high frequency of cage-mate barbering in *Btbd3* KO colony home cages (Fig. 1a); therefore, we monitored barbering behavior by pair-housing mice of identical sex and genotype. Chi-square analysis revealed that barbering was unevenly distributed across the three genotypes (Fig. 1b; *Χ*^2^_(2, *n*=647)_ = 11.38; *p* < 0.005). *Btbd3* HT (*Χ*^2^_(1, *n*=486)_ = 7.73; *p* < 0.01) and KO (*Χ*^2^_(1, *n*=332)_ = 11.77; *p* < 0.0001) groups each had an increased incidence of barbering compared to WT. A bootstrap analysis (100,000 iterations) confirmed that all of the chi-square statistics above were unlikely (*p*’s < 0.05) to be due to the uneven group numbers that resulted from heterozygous breeding in this observational cohort. Bald patches in the fur were observed primarily on the dorsal surface of the body and the face, characteristic of barbering by cage mates, rather than self-barbering^33^.

We then assessed the effects of OCD-effective (SRI fluoxetine) or OCD-ineffective antidepressant treatment (NRI desipramine) on barbering^36,37^ for 14 weeks to determine whether barbering may be a compulsive-like phenotype. Pooled across genotypes, log-rank tests revealed that fluoxetine reduced the onset of barbering relative to vehicle starting at 4 weeks of treatment (Fig. 1c), and throughout the remainder of treatment, except for weeks 7 and 8 (Supplementary Information). In contrast, desipramine did not affect onset of barbering (*Χ*^2^_(1, *n*=177)_ = 2.09; *p* = 0.15). Furthermore, within fluoxetine-treated animals, a genotype effect was identified (*X*^2^_(2, *n*=95)_ = 16.75; *p* < 0.0005). Therefore, the effect of fluoxetine on barbering within each genotype was examined. Within *Btbd3* WT mice, fluoxetine prevented barbering onset (Fig. 1d), and this effect was significant by 6 weeks of treatment (*Χ*^2^_(1, *n*=56)_ = 4.59; *p* < 0.05). Within *Btbd3* HT mice, fluoxetine became effective at 12 weeks of treatment (Fig. 1e; *Χ*^2^_(1, *n*=64)_ = 4.50; *p* < 0.05) and remained significant through week 14 (*Χ*^2^_(1, *n*=64)_ = 4.50; *p* < 0.05). In contrast, fluoxetine never affected *Btbd3* KO barbering incidence (Fig. 1f; week 14: *Χ*^2^_(1, *n*=65)_ = 0.40; *p* = 0.53). These effects were not due to genotypic differences in fluoxetine metabolism (Supplementary Information).

Next, we tested cognitive domains relevant to compulsive disorders using operant conditioning. ANOVAs revealed that there was no effect of genotype on FR1 training (*F*_(2,49)_ = 0.27; *p* = 0.76) or on false alarm rate in the Go/No-Go task (Supplementary Information), which measures response inhibition^39,53^. In the PLT, which measures reinforcement learning and response strategies^41^, no effects of genotype were identified for Block 1 (reward probabilities of 90%/10%) or Block 2 (reward probabilities of 80%/20%; data not shown). However, in Block 3 (reward probabilities of 70%/30%), *Btbd3* KO mice were less accurate than WT or HT mice (Fig. 1g; *F*_(2,35)_ = 8.01; *p* < 0.005). An ANOVA and post hoc tests revealed a higher proportion of target lose-shift responses in *Btbd3* KO mice versus WT mice (*F*_(2,35)_ = 4.96; *p* < 0.05; Fig. 1h). Furthermore, *Btbd3* KO mice more frequently reselected the non-target port after being rewarded than WT mice (Fig. 1i; *F*_(2,23)_ = 4.24; *p* < 0.05). Genotype did not affect target win-stay or non-target lose-shift patterns (Supplementary Fig. 1d, e). Lastly, mice were assessed in the PRBP test for any potentially confounding effects of genotype on motivation in the PLT, but genotype did not affect breakpoint (Fig. 1j; *F*_(2,43)_ = 1.58; *p* = 0.22).

Next, wheel-running was assessed, which is excessive in some rodent models of compulsive behavior^54^. A three-way interaction of day, cycle, and genotype was identified by a repeated measures ANOVA for wheel revolutions (*F*_(12,312)_ = 2.32; *p* < 0.01). Post hoc ANOVAs and then post hoc tests revealed that *Btbd3* KO mice accrued more wheel revolutions than WT or HT mice during the dark cycle (Fig. 1k; *F*_(2,55)_ = 4.57; *p* < 0.05), but not the light cycle (*F*_(2,55)_ = 0.35; *p* = 0.70; data not shown).

### *Btbd3* KO mice show reduced exploration

**Fig. 2:**
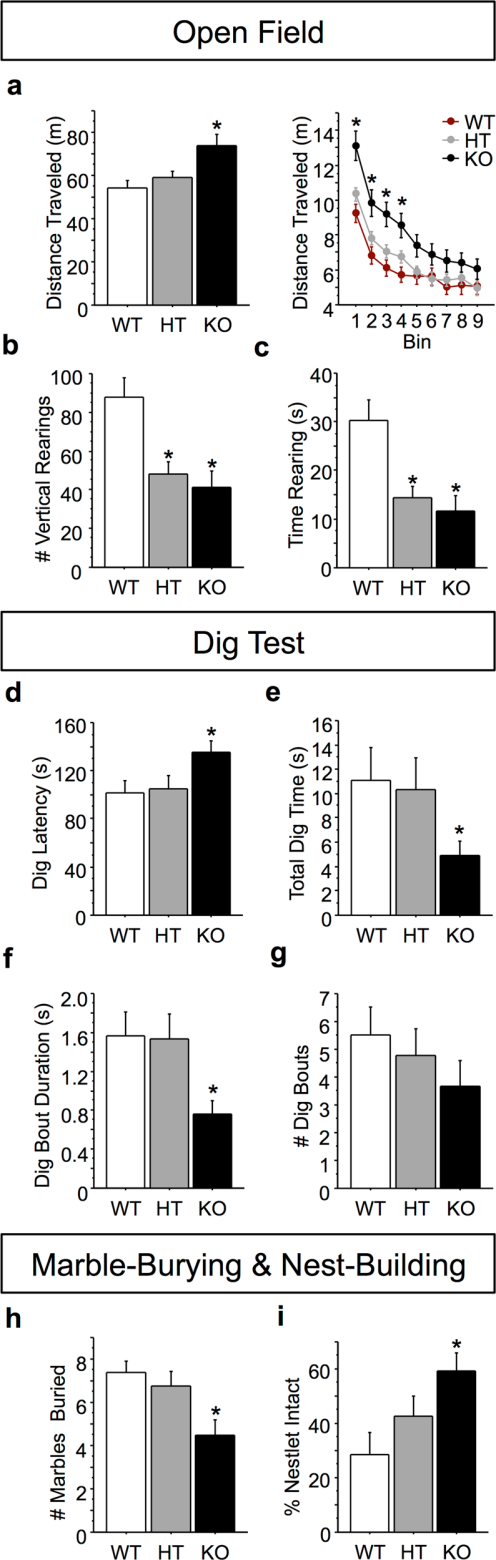
*Btbd3* knockout reduces exploration. **a** *Btbd3* KO mice traveled a greater total distance in the OF (*n* = 17 female/27 male WT, 34 female/27 male HT, and 11 female/20 male KO mice) than HT or WT mice overall (left panel), which was confined to the first 20 min of testing as revealed by the 5-min bin analysis (right panel). **b** *Btbd3* HT and KO mice showed fewer instances of vertical rearing and **c** spent less time rearing across the session than WT mice in the OF. **d**–**g** In the dig test (*n* = 13 female/14 male WT, 15 female/16 male HT, 20 female/13 male KO mice), *Btbd3* KO mice had a greater latency to initiate digging behavior **d**, reduced total time spent digging **e**, and dug in briefer bouts than controls **f**. The number of dig bouts in the dig test was unaffected by *Btbd3* genotype **g**. **h** *Btbd3* KO mice buried fewer marbles (*n* = 14 female/12 male WT, 12 female/12 male HT, 9 female/13 male KO mice) than WT mice in the marble-burying test. **i** *Btbd3* KO mice left a greater portion of the nestlet intact than WT mice in the 8-h nest-building paradigm (*n* = 12/genotype/sex). Results are expressed as mean values ± SEM. *p < 0.05 vs. WT group in ANOVAs resolved with post hoc tests. WT wild-type, HT heterozygous, KO knockout, OF open field

We next assessed exploration in *Btbd3* mice, as novelty-seeking is reduced in OCD^55–58^. In the OF test, a genotype by bin interaction (*F*_(16,1040)_ = 2.57; *p* < 0.0001) in a repeated measures ANOVA and post hoc tests revealed that *Btbd3* KO mice traveled a greater total distance than WT or HT mice in bins 1–4 (Fig. 2a). All genotypes exhibited locomotor habituation (Supplementary Information). *Btbd3* HT and KO mice had reduced instances of vertical rearing relative to WT mice (Fig. 2b; *F*_(2,130)_ = 6.97; *p* < 0.005) and spent less time rearing than WT mice (Fig. 2c; *F*_(2,130)_ = 7.18; *p* < 0.005).

*Btbd3* mice were then assessed for exploratory digging. The distribution of the data violated the normality assumption for using parametric tests even after attempts at transforming the data, so nonparametric statistics were used. A Kruskal–Wallis one-way ANOVA found a main effect of genotype on latency to dig (*H*_(2)_ = 6.43; *p* < 0.05), with *Btbd3* KO mice showing a longer latency to dig than WT mice (Fig. 2d; *U* = 289; *p* < 0.025). Furthermore, *Btbd3* KO mice spent less time digging than WTs (Fig. 2e; *H*_(2)_ = 5.93; *p* < 0.05; *U* = 286; *p* < 0.025). Lastly, genotype altered bout duration (*H*_(2)_ = 8.20; *p* < 0.05), with *Btbd3* KO mice performing briefer digging bouts than WT mice (Fig. 2f; *U* = 265; *p* < 0.025). No effect of genotype was identified for number of digging bouts (Fig. 2g; *H*_(2)_ = 4.36; *p* = 0.11).

*Btbd3* mice were tested in the marble-burying paradigm to confirm genotype effects on digging behavior^44–47^. An ANOVA and post hoc tests revealed that *Btbd3* KO mice buried fewer marbles than WT or HT mice (Fig. 2h; *F*_(2,66)_ = 5.56; *p* < 0.01).

Nest-building is considered a measure of well-being in mice^48^, and nest-building deficits have been found in numerous neuropsychiatric disorder models^59–62^. In the nest-building task, a main effect of genotype was identified for original nestlet percentage remaining intact in an ANOVA (*F*_(2,65)_ = 4.33; *p* < 0.05), with post hoc tests showing that *Btbd3* KO mice left more nestlet untouched than WT mice (Fig. 2i).

### *Btbd3* does not modulate anxiety-like or depression-like behaviors or basic sensory and motor functioning

**Fig. 3:**
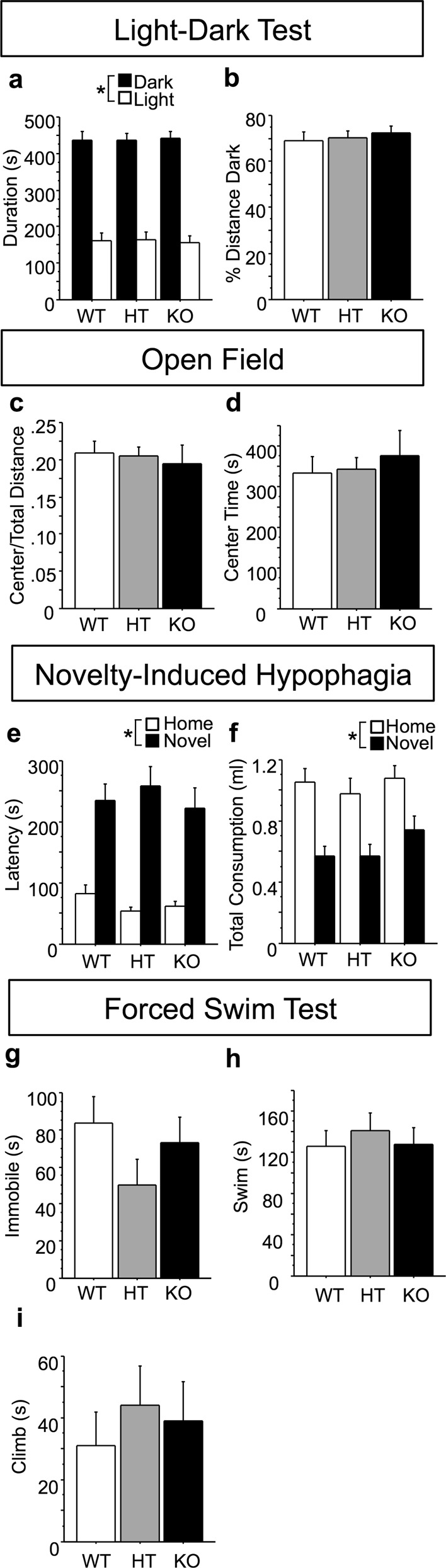
BTBD3 does not affect anxiety-like or depression-like behavior. *Btbd3* genotype had no effect on proportion of time spent **a** or distance traveled **b** in the dark side of the light-dark box (*n* = 15/genotype/sex), proportion of distance traveled **c** or time spent in the center **d** of the OF (*n* = 17 female/27 male WT, 34 female/27 male HT, and 11 female/20 male KO mice), total latency to drink **e**, or total consumption **f** of sweetened condensed milk in the NIH (*n* = 10 each female/male WT, 10 female/9 male HT, 9 each female/male KO mice), time spent immobile **g**, swimming **h**, or climbing **i** in the FST (*n* = 10/genotype/sex, except female WT *n* = 9). Results are expressed as mean values ± SEM. *p < 0.05 between conditions (dark versus light; home versus novel cage) as determined by ANOVAs. OF open field, NIH novelty-induced hypophagia, FST forced swim test

Anxiety and depression are often comorbid with OCD^2,63^. Thus, *Btbd3* mice were assessed for anxiety-like and depression-like behaviors. In the light-dark test, no effects of genotype were identified for either duration on the light versus the dark side of the chamber (Fig. 3a) or percent distance traveled in the dark side (Fig. 3b). An ANOVA revealed a main effect of side of the chamber for duration (*F*_(1,84)_ = 140.97; *p* < 0.0001), indicating that animals spent more time on the dark side. In the OF test, no effect of genotype was identified for proportion of distance traveled in the center (Fig. 3c) or time spent in the center (Fig. 3d). Mice were assessed in the NIH test as an additional measure of anxiety-like behavior. Since latency data are often skewed, we examined the distribution of the data, and as a result log-transformed latency to drink for statistical analysis. Neither a main effect of genotype nor an interaction between genotype and cage condition was identified for latency to drink (Fig. 3e). A main effect of cage condition on latency to drink was identified (*F*_(1,49)_ = 152.30; *p* < 0.0001), indicating a longer latency to consume the sweetened condensed milk in the novel cage. No effects of genotype were found for total consumption (Fig. 3f). A main effect of cage condition was identified for total consumption (*F*_(1,51)_ = 102.50; *p* < 0.0001), indicating reduced consumption of sweetened condensed milk in the novel versus the home cage.

*Btbd3* mice were assessed in the FST to evaluate depression-like behavior. No effect of genotype was identified for time spent immobile (Fig. 3g), swimming (Fig. 3h), or climbing (Fig. 3i).

Finally, basic sensorimotor functioning was assessed in *Btbd3* mice to screen for potentially confounding effects on compulsive-like and exploratory behaviors. No effects of genotype were found in auditory, olfactory, somatosensory, or motor function (Supplementary Fig. 1f, g; 2). See Supplementary Information.

### Effects of *Btbd3* expression on limbic CSTC dendritic morphology

Dendritic morphology was assessed in limbic CSTC brain regions with dense *Btbd3* expression because BTBD3 was previously reported to play a role in dendritic morphology^16^. No effects of genotype were identified for dendritic branching or spine density in the CA1 or dentate gyrus subregions of hippocampus, or in the mediodorsal thalamus. However, increased spine density in *Btbd3* KO mice was identified in the ACC (Supplementary Fig. 3). See Supplementary Information.

### Whole hippocampal *Btbd3* KD induces compulsive-like behavior and decreases exploration

**Fig. 4:**
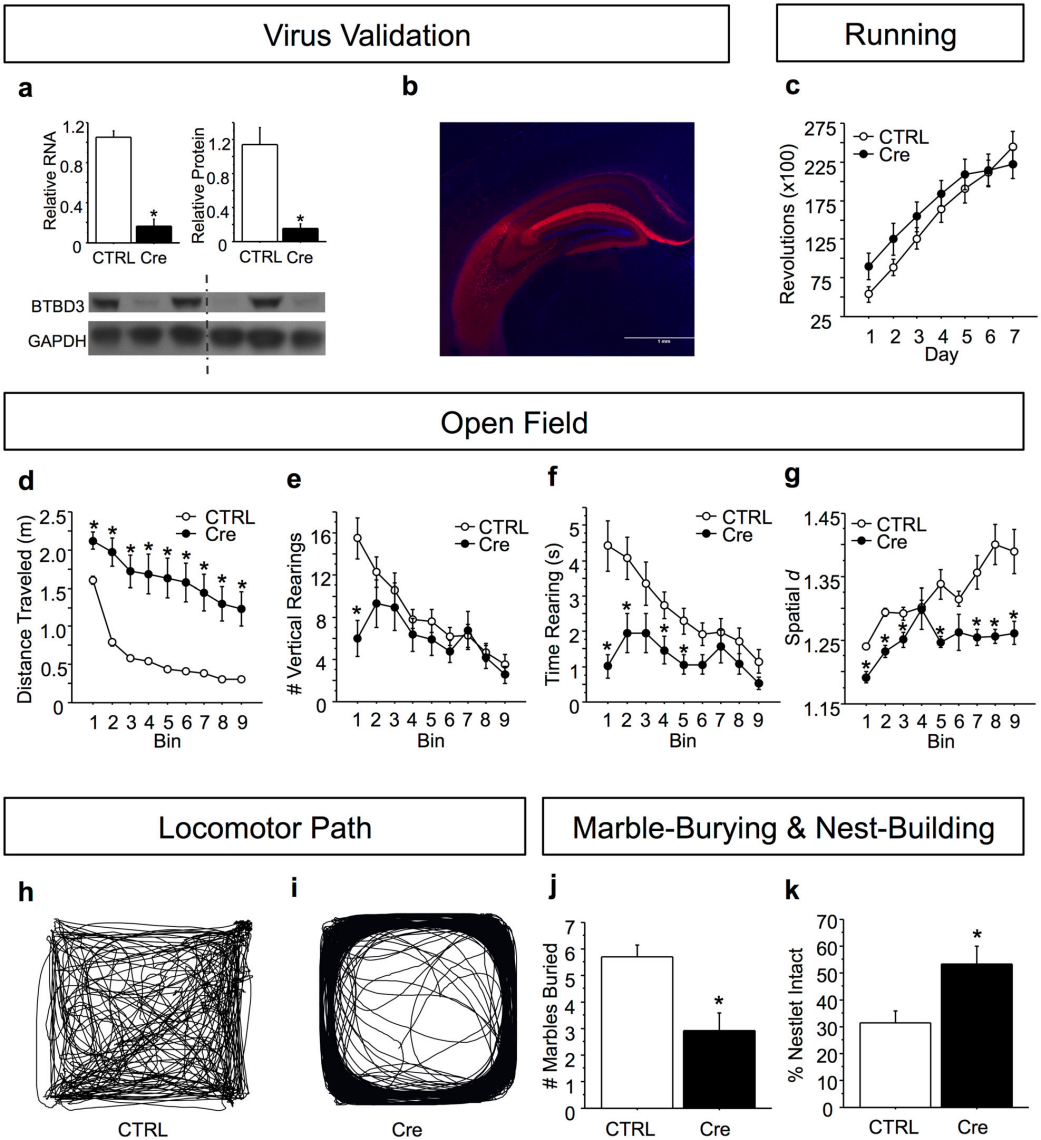
Neonatal *Btbd3* knockdown in whole hippocampus recapitulates the *Btbd3* knockout phenotype. Cre virus successfully knocked down RNA (**a**, top left) and protein (**a**, top right; bottom) in hippocampus in a qPCR assay (*n* = 7 AAV-tdTomato, 2 AAV-Cre virus) and a western blot (*n* = 3/virus) relative to CTRL, respectively. The western blot image (**a**, bottom) shows samples from CTRL virus-infused mice in lanes 1, 3, and 5, and samples from *Btbd3* KD mice in lanes 2, 4, and 6. The dotted line indicates that the image was interrupted between these lanes in order to remove space between the lanes, but the left and right-hand sides are from the same image of the blot. **b** Representative example of viral reporter tdTomato expression in hippocampus. **c** Hippocampal *Btbd3* KD did not affect number of wheel revolutions in the voluntary home cage wheel-running assessment (*n* = 19 female/25 male AAV-tdTomato and 15 female/12 male AAV-Cre infused mice). **d** Hippocampal *Btbd3* KD increased distance traveled in the OF throughout testing (*n* = 19 female/26 male AAV-tdTomato and 16 female/13 male AAV-Cre infused mice). **e** Hippocampal *Btbd3* KD reduced instances of vertical rearing only within the first 5-min bin. **f** Hippocampal *Btbd3* KD reduced time spent rearing in the first half of testing. **g** Hippocampal *Btbd3* KD reduced spatial *d* throughout testing (example traces in **h**, **i**). Hippocampal *Btbd3* KD mice buried fewer marbles in the marble-burying paradigm (*n* = 19 female/26 male AAV-tdTomato and 16 female/13 male AAV-Cre infused mice) **j** and left more nestlet unused in the overnight nest-building test **k** than CTRL mice (*n* = 19 female/26 male AAV-tdTomato and 16 female/13 male AAV-Cre infused mice). Results are expressed as mean values ± SEM. *p < 0.05 vs. CTRL group in ANOVAs resolved with post hoc tests. KD knockdown, CTRL control virus: AAV-tdTomato, OF open field, AAV adeno-associated virus, qPCR quantitative polymerase chain reaction, Cre AAV-Cre virus

Due to robust *Btbd3* expression in hippocampus^17^ and the role of hippocampus in several behavioral phenotypes for which deficits were identified in *Btbd3* KO mice^45,64^, we assessed the effects of neonatal, whole hippocampal *Btbd3* KD on behavior during adulthood. We confirmed KD of *Btbd3* RNA (Fig. 4a, top left panel; *F*_(1,7)_ = 46.39; *p* < 0.0005) and protein (Fig. 4a, top right panel; *F*_(1,4)_ = 22.10; *p* < 0.01) in the hippocampus of *Btbd3*^flox^ mice receiving viral infusion of AAV-Cre versus AAV-tdTomato. *Btbd3* KD did not alter wheel-running (Fig. 4c), but increased locomotor activity (Fig. 4d; *F*_(1,70)_ = 48.85; *p* < 0.0001) and reduced locomotor habituation in the OF (Supplementary Information). *Btbd3* KD mice had reduced instances of rearing within the first 5 min (Fig. 4e; *F*_(8,560)_ = 3.93; *p* < 0.0005). For time spent rearing (Fig. 4f), an interaction between viral condition and bin (*F*_(8,560)_ = 3.49; *p* < 0.001) and post hoc tests revealed that *Btbd3* KD mice spent less time rearing than control mice in time bins 1, 2, 4, and 5. *Btbd3* KD mice exhibited reductions in spatial *d* within each time bin (Fig. 4g), except bins 4 and 6 (Fig. 4h, i; *F*_(8,504)_ = 2.32; *p* < 0.05).

*Btbd3* KD mice buried fewer marbles than control mice in the marble-burying test (Fig. 4j; *F*_(1,70)_ = 10.87; *p* < 0.005) and used less nestlet than control mice in the nest-building test (*F*_(1,70)_ = 6.47; *p* < 0.05) as determined by an ANOVA followed by post hoc tests (Fig. 4k).

### Dorsal hippocampal *Btbd3* KD does not mimic the *Btbd3* KO phenotype

**Fig. 5:**
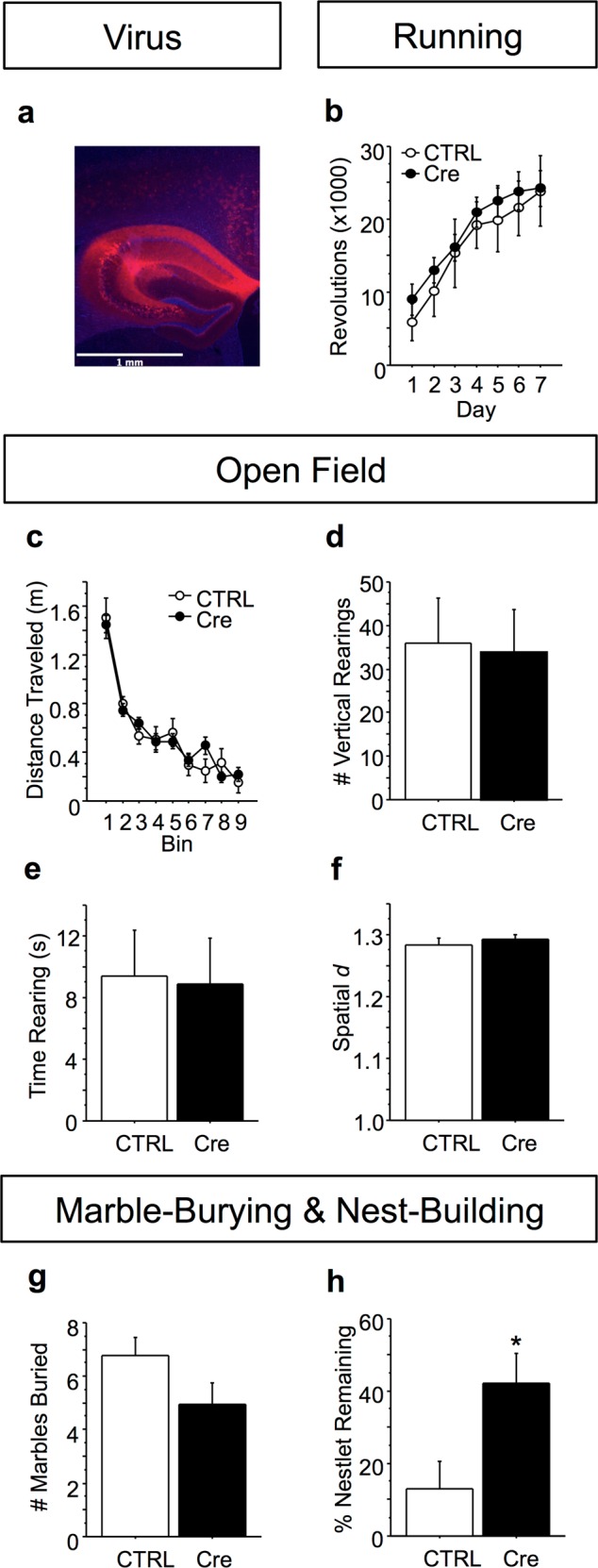
Neonatal *Btbd3* knockdown in dorsal hippocampus does not recapitulate the *Btbd3* knockout phenotype. **a** Example of viral reporter tdTomato expression in dorsal hippocampus. **b** Dorsal hippocampal *Btbd3* KD did not affect number of revolutions in wheel-running (*n* = 5 female/4 male AAV-tdTomato, 9 female/9 male AAV-Cre mice), **c** distance traveled in the OF (*n* = 5 female/4 male AAV-tdTomato, 11 female/9 male AAV-Cre mice), vertical rearing in the OF **d**, **e**, spatial *d* in the OF **f**, or **g** marble-burying (*n* = 5 female/4 male AAV-tdTomato, 11 female/9 male AAV-Cre mice). **h** Neonatal dorsal hippocampal *Btbd3* KD mice left more nestlet unused than CTRL mice in the nest-building paradigm (*n* = 5 female/4 male AAV-tdTomato, 11 female/9 male AAV-Cre mice). Results are expressed as mean values ± SEM. *p < 0.05 vs. CTRL group in ANOVAs resolved with post hoc tests. KD knockdown, CTRL control virus: AAV-tdTomato

Next, we investigated whether *Btbd3* KD in dorsal hippocampus, which was recently implicated in goal-directed planning^26^, would be sufficient to induce the *Btbd3* behavioral profile. No effect of *Btbd3* KD was identified on wheel-running (Fig. 5b), distance traveled (Fig. 5c), instances or time spent rearing (Fig. 5d, e), spatial *d* (Fig. 5f), or marble-burying (Fig. 5g; *F*_(1,25)_ = 1.85; *p* = 0.19) in ANOVAs. The only effect of neonatal *Btbd3* KD in dorsal hippocampus was impaired nest-building in the nest-building test (Fig. 5h; *F*_(1,25)_ = 4.58; *p* < 0.05).

## Discussion

Our results show that BTBD3 regulates compulsive-like and exploratory behaviors across multiple paradigms in mice. *Btbd3* HT and KO mice exhibited increased barbering, which was reduced by effective (SRI), but not ineffective (NRI), OCD treatment. Additionally, *Btbd3* KO mice exhibited excessive wheel-running and poor goal-directed decision-making, a trait that is thought to underlie compulsive behavior. *Btbd3* HT and KO mice were less exploratory in the OF, dig, and marble-burying tests. Furthermore, whole hippocampal, but not dorsal, neonatal *Btbd3* KD increased compulsive-like behaviors and reduced exploration during adulthood, largely mimicking the phenotype of constitutive *Btbd3* KO mice. Surprisingly, *Btbd3* knockout spared dendritic morphology within hippocampal subregions, but increased spine density in ACC layer II/III pyramidal neurons, suggesting that an alternative function of *Btbd3* may mediate the hippocampus-dependent behavioral effects observed. Our findings indicate that reduced hippocampal *Btbd3* expression leads to the development of compulsive-like behaviors and reductions in exploration.

*Btbd3* HT and KO mice exhibited compulsive-like behaviors indicated by increased barbering, wheel-running, and impaired goal-directed behavior. Barbering is an abnormal behavior observed only in confined animals^38^, and is considered a repetitive, compulsive-like behavior^33,54,65^. The idea that barbering is a compulsive-like behavior is reinforced by our finding that chronic SRI, but not NRI, treatment reduced barbering, paralleling treatment response in OCD^8^. Interestingly, our finding that barbering incidence was reduced by chronic fluoxetine treatment in *Btbd3* WT and HT, but not KO mice suggests that *Btbd3* expression may be required for these anti-compulsive effects of fluoxetine. Increased barbering has previously been reported in other genetically altered mouse lines exhibiting a compulsive-like phenotype^66^, and has been associated with deficits in extradimensional shifting^65^, which measures cognitive flexibility^7^ and is deficient in OCD^67–69^. While excessive wheel-running has also been proposed as compulsive-like in rodent food restriction-induced hyperactivity models based on the pharmacological response profile^70,71^, effects across studies are not fully consistent with OCD^72^. Thus, the relationship between excessive wheel-running and compulsivity appears somewhat unclear^73^. Alternatively, other mouse lines exhibiting a compulsive-like phenotype have also reported increased barbering and excessive wheel-running^54^, suggesting that these phenotypes may be linked. In addition to increased barbering and wheel-running, *Btbd3* KO mice also exhibited impaired decision-making in the PLT, which assesses the balance of goal-directed versus habitual decision-making strategies during reinforcement learning^41,74^. Compulsive behavior has been associated with alterations in this balance, with a shift toward more habitual, and less goal-directed tendencies^3,4^, as observed in OCD patients^28–30^. Thus, goal-directed learning impairments are thought to contribute to the development of compulsivity^31,32^. Therefore, the reduction in goal-directed decision-making we observed in *Btbd3* KO mice is consistent with the increases in barbering and wheel-running we also observed in these mice. That *Btbd3* KO mice exhibit multiple different compulsive-like phenotypes is consistent with evidence that discrete types of repetitive behaviors are interrelated and subserved by partially overlapping CSTC circuitry^19^.

*Btbd3* HT and KO mice also exhibited reduced exploratory drive. Robust reductions in vertical rearing were found in *Btbd3* HT and KO mice. *Btbd3* KO mice also showed reduced digging^43^ and marble-burying. While increased marble-burying has been widely used as a measure of anxiety-like or compulsive-like behavior, this behavior is reduced by many compounds that are ineffective treatments for anxiety or compulsivity^73,75^, including acute treatment with SRIs^76^. Furthermore, several rodent models of abnormal repetitive behavior exhibit reductions in marble-burying, concomitant with reduced digging or vertical rearing behavior^77–79^. Thus, marble-burying has also been suggested to measure exploration^44–46^. Interestingly, exploration is an integral component of goal-directed behavior^80^, suggesting that these two impairments in *Btbd3* KO mice might be related. Moreover, novelty-seeking, a trait that drives exploration, is reduced in patients with OCD^55–58^. Lastly, *Btbd3* KO mice also showed impaired nest-building, which has been associated with reduced well-being^48^.

Interestingly, anxiety-like and depression-like behaviors were unaltered in *Btbd3* HT and KO mice. Anxiety-like behavior was unaffected in the light-dark, OF, and NIH tests, well-established anxiety measures in rodents. These results strongly suggest that reduced exploration in *Btbd3* HT and KO mice does not reflect augmented anxiety, consistent with previous reports that exploration and anxiety-like behavior are dissociable^81^. Furthermore, depression-like behavior was unaffected by genotype in the FST. Thus, our findings suggest that the behavioral phenotypes observed in *Btbd3* KO mice are not modulated by anxiety-like or depression-like states. While anxiety and depression are highly comorbid with OCD^2,63^, anxiety and affective disorders are distinct from OCD. For example, traits characteristic of OCD, eating disorders, and addiction correlate with goal-directed behavior deficits in humans, whereas anxiety and depression do not^3^, suggesting distinct neuropsychological endophenotypes between these classes of disorders. Moreover, SRIs and NRIs serve as effective treatment for anxiety and depression^82–85^, whereas only SRIs provide effective monotherapy in OCD^8^, a treatment profile that parallels our barbering results. This distinction is further reflected in the DSM-5, which removed OCD from the anxiety disorders category and does not include anxiety as a core symptom of OCD^1^. Thus, our results suggest that BTBD3 may modulate neural processes contributing to compulsive-like behaviors, including impaired exploratory drive and/or reduced goal-directed behavior, which are independent of anxious-depressive phenotypes. The *Btbd3* behavioral profile is thus distinct from several genetic mouse models of OCD, which exhibit a repetitive behavior, such as excessive self-grooming or self-barbering concomitant with increased anxiety-like behaviors^66,86,87^.

We did not observe effects of *Btbd3* genotype on additional behavioral measures including motivation, startle, or sensorimotor behaviors. Motivation was unaltered in *Btbd3* HT and KO mice in the PRBP, as was response inhibition as indicated by false alarm rate in the Go/No-Go task (Supplementary Fig. 1b). *Btbd3* HT and KO mice also did not show differences in sensorimotor gating (Supplementary Fig. 1f) or startle reactivity (Supplementary Fig. 1g). Furthermore, no effects of *Btbd3* genotype were observed on sensory or motor measures in the olfactory dis/habituation or olfactory memory tests, the whisker-brushing test, or the footprint test (Supplementary Fig. 2a–i). Young adult *Btbd3* KO mice had modestly reduced bodyweight, which was unlikely to confound the other tests performed (Supplementary Fig. 2j): although, we note the common genetic variation overlap between OCD and anorexia nervosa, and the negative genetic correlation of both with body mass index^88^. Lastly, no effects of sex were found for the phenotypic differences identified.

Neonatal *Btbd3* KD in the whole hippocampus induced compulsive-like behavior and reduced exploration during adulthood, mirroring the phenotype observed in constitutive *Btbd3* KO mice, with minor exceptions. Although both models showed exploration deficits, increased locomotion in the OF, and increased compulsive-like behavior, hippocampal *Btbd3* KD mice showed decreased spatial *d* but unaltered wheel-running, while constitutive *Btbd3* KO mice showed the opposite pattern. These discrepancies may reflect different compensatory changes or distinct *Btbd3* expression patterns. We did not assess barbering in the *Btbd3* KD cohorts due to the large sample sizes required for these observations. Interestingly, a pharmacological mouse model of aspects of OCD shows a highly similar behavioral profile, in which 5-HT1B receptor stimulation induces hyperactivity, low spatial *d*, reduced vertical rearing, reduced exploratory digging, and impaired delayed alternation performance^10,11,35,89^. This observation further suggests that reduced species-typical exploratory behaviors may be causally related to increased compulsive-like behaviors. Importantly, neonatal *Btbd3* KD in the dorsal half of the hippocampus did not produce any of the behavioral effects of whole hippocampal *Btbd3* KD, except for nest-building deficits. Altogether, our findings suggest that *Btbd3* KD in the whole, but not dorsal, hippocampus induces compulsive-like behavior and reductions in exploration.

Surprisingly, *Btbd3* genotype altered dendritic morphology in ACC layer II/III pyramidal neurons, but not in the mediodorsal thalamus, or CA1 or dentate gyrus of the hippocampus (Supplementary Fig. 3). This result was unexpected given the important role of BTBD3 in dendritic morphology in the developing barrel cortex^16^, and suggests that additional region-specific functions of BTBD3, or compensatory changes in constitutive *Btbd3* KO mice produced the observed phenotypes. For instance, BTBD3 interacts with cell signaling proteins, such as SEC24C, which is required for trafficking the serotonin transporter to the cell membrane^90,91^, and CLTC, clathrin heavy chain protein, which is integral to synaptic transmission^90,92^. Only ACC layer II/III pyramidal neurons had increased spine density in *Btbd3* KO mice, which might reflect alterations in the hippocampus-to-ACC circuit, which is implicated in goal-directed behavior^93^. Indeed, neonatal ventral hippocampal lesions in rodents are thought to disrupt the development of prefrontal circuitry^94^ and enhance mesolimbic dopamine signaling^95^, resulting in extradimensional shifting deficits, hyperactivity, increased apomorphine-induced stereotypy, and reduced rearing, marble-burying, and habituation to novelty^96–99^. This behavioral profile is similar to the phenotype of *Btbd3* KO and hippocampal *Btbd3* KD mice, and aligns with the minimal behavioral effects of dorsal hippocampus-specific *Btbd3* KD. While speculative, our results suggest the possibility that loss of BTBD3 primarily in the ventral hippocampus may produce the observed phenotype through disruption of CSTC circuitry.

To our knowledge, our results comprise the first evidence for a role of *Btbd3* in behavior, which is to modulate compulsive-like and exploratory behaviors in mice. Our results support the possibility that the SNP rs6131295, an eQTL for *BTBD3*, may be of relevance to neuropsychiatric disorders, despite only exceeding the genome-wide significance threshold in the trio portion of an OCD GWAS^13^. Indeed, OCD patients exhibit compulsive behaviors, goal-directed behavior deficits^28–30^, and reduced novelty-seeking^55–58^. However, other neuropsychiatric disorders exhibit one or more of these phenotypes, including autism spectrum disorder^100,101^, addiction^4^, and binge-eating disorder^4^, suggesting the potential relevance of BTBD3 to other psychiatric disorders. Furthermore, SNPs revealed by GWASs likely lead to subtle changes in gene expression, in comparison to the large reductions in *Btbd3* expression studied here in mice. Yet, risk genes for neuropsychiatric disorders identified by GWAS have been suggested to show an increased burden for rare variants with larger damaging effects^102,103^.

Our findings show that reduced *Btbd3* expression induces compulsive-like behaviors and reduced exploration, phenotypes highly relevant to neuropsychiatric disorders including OCD. Furthermore, hippocampal *Btbd3* expression plays an essential role in the regulation of these behaviors, but loss of BTBD3 in dorsal hippocampus is not sufficient to induce these effects. Future work will determine the molecular mechanisms and developmental time window in which *Btbd3* expression confers these phenotypes. As any behavioral role of *Btbd3* was previously unreported, our work highlights the importance of investigating unknown genes identified in an unbiased fashion^104^.

## Supplementary information

Supplementary Information.

Supplementary Figures.
