# Supplementary material for: Btbd3 expression regulates compulsive-like and exploratory behaviors in mice
Source: Transl Psychiatry. 2019 Sep 9;9:222. doi: 10.1038/s41398-019-0558-7 (PMC6733800; doi:10.1038/s41398-019-0558-7)
Supplement: Supplementary file 1 — Supplementary Information. [file 41398_2019_558_MOESM1_ESM.pdf]

**SUPPLEMENTARY INFORMATION**

*Btbd3* expression regulates compulsive-like and exploratory behaviors in mice

Summer L Thompson PhD, Amanda C Welch BA, Emily V Ho BS, João M Bessa MD PhD, Carlos Portugal-Nunes BS, Mónica Morais PhD, Jared W Young PhD, James A Knowles MD PhD, Stephanie C Dulawa PhD

## METHODS

*Barbering*. Barbering was assessed weekly, and identified by visual observation of bald patches in the fur or clipped whiskers. Mice were photographed weekly to record the barbering phenotype. In the treatment study, drug groups were age-matched, and no subjects had barbered prior to initiation of the study. Cumulative barbering was assessed using Kaplan-Meier curves. Onset of barbering behavior served as the categorical, cumulative outcome measure for survival.

*Go/No-Go*. Male *Btbd3* WT, HT, and KO mice were food-restricted to 85% of baseline bodyweight. Training and testing occurred in five-choice operant chambers (Med Associates) equipped with 5 nose poke ports with recessed LEDs, an additional light over the central nose poke port, a house light, and a food delivery magazine on the opposite wall from the nose poke ports. Responses in the nose poke ports and magazine were detected using infrared beams. Liquid reinforcement was administered using peristaltic pumps (Lafayette Instruments, Lafayette, IN, USA) and consisted of strawberry milkshake (Nesquik mixed with nonfat milk) in 30 µl increments. Tasks were programmed in MED-PC for Windows (Med Associates).

Mice were initially trained to retrieve reward from the magazine (Phase I). The magazine was lit and administered reward every 15 seconds in a 20-minute session. Once mice retrieved 60 rewards per session, they moved on to the subsequent training phase. In Phase II, mice were trained to respond to the

central nose poke port on a fixed-ratio 1 schedule in a 30-minute session. The central nose poke port was lit continuously until the animal nose-poked for reward. Once the mouse retrieved the reward, a variable intertrial interval (ITI) was utilized. Once animals consistently responded in the central port, they were trained in the Go/No-Go paradigm. In Phase III of training, mice were trained to respond in the central port during a timed stimulus presentation, in which the central port was lit and active for 8 seconds, then 5 seconds, then finally 3 seconds, in five, 30-minute sessions for each stimulus duration. Mice that did not reach criterion (20 successful trials within a session) by the end of the fifth training session at the 3-second stimulus interval were excluded from analysis for the Go/No-Go paradigm. Mice were also punished for incorrect responses (responses in unlit ports), for omissions (lack of response during the stimulus window), and for premature responses (responses prior to initiation of the trial indicated by light in the central port). Punishment consisted of a 4 second time out with the house light on. Animals had to retrieve reward from the magazine to initiate the subsequent trial. Initially, a variable ITI of 5 - 17 seconds was used. However, this resulted in high levels of premature responding; thus the ITI was shortened to 3 - 7 seconds during Phase III of training and was maintained throughout Go/No-Go testing.

Mice then underwent 15 30-minute Go/No-Go test sessions. A "Go" trial consisted of the central port being lit during the 3-second stimulus presentation, as during training except that responses in incorrect ports were not punished. "No-Go" trials consisted of the central port being lit concomitantly with an additional, green, light just above the central port for the 3-second stimulus interval, creating a compound stimulus. Mice were rewarded for refraining from responding to the compound stimulus, and were punished with a time out for responding (a "false alarm"). The first five days were training for the No-Go stimulus. The remaining ten days of testing were used for analysis. The false alarm rate provided the primary outcome measure of response inhibition, with a high false alarm rate indicating impairments in action restraint, a type of impulsivity[1]. Premature responding provided the secondary response inhibition measure. The sensitivity index d-prime (d') measures how well animals learn the task by comparing the hit rate and false alarm rate.

*Progressive Ratio Breakpoint*. Following completion of Go/No-Go testing, mice moved on to the PRBP task. First, mice underwent a refresher training session at Phase II. The next day, mice were tested in the 60-minute PRBP test session, as previously described[2]. Briefly, mice were required to respond in the central nose poke port progressively more times in order to earn a reward in the following steps: 1, 2, 4, 7, 11, 16, 22, 29, 37, 46, 56 and 67 responses required for reward. The "breakpoint," defined as the highest number of responses the animal would perform to obtain a reward, was the primary outcome measure. All mice that reached criterion for Go/No-Go testing, regardless of the days to criterion, were included in the PRBP and PLT analyses, as days to criterion had no effect on breakpoint ($F_{(2,43)} = 1.03$; $p = .37$).

*Probabilistic Learning Task*. Finally, animals underwent PLT testing. First, animals underwent one day of training using a modified version of the Phase II training session, in which all five ports were lit and active for earning reward. The following day, mice underwent PLT testing in 3 blocks of 60 trials for a maximum 60-minute session. Two ports were lit for each trial, and were counterbalanced across groups. Within a block, one port was the "target" port and the other the "non-target." The target port was commonly rewarded (90, 80, or 70% of the trials for blocks 1, 2, and 3 respectively) and the non-target port was uncommonly rewarded (10, 20, or 30% of the trials). After completing the 60 trials for block 1 (90 or 10% reward probabilities), the two previously lit ports were extinguished and two new ports were lit, one with 80% and the other with a 20% reward probability. Similarly, for the third block, the previously lit ports were extinguished and two new ports were lit, one with 70% and one with 30% reward probability. "Win-stay" and "lose-shift" strategies on the target and non-target ports were the primary outcome measures, where "win-stay" denotes a trial in which an animal returns to the same port in which they were rewarded on the previous trial. "Lose-shift" refers to a trial in which an animal shifts responding to the opposite port after receiving punishment on the previous trial. Accuracy was also a primary outcome measure, defined as the percentage of trials in which the animal responded on the target port.

*Prepulse Inhibition*. Prepulse inhibition (PPI) was assessed as previously described[3]. In brief, mice were placed in startle chambers (San Diego Instruments, San Diego, CA, USA). Startle response amplitude was measured for startle alone (120 dB), prepulse trials (3, 6, or 12 dB prepulse followed by 120 dB pulse), or no stimulus. PPI was calculated as 100 * (startle - prepulse)/startle amplitude.

*Olfactory Dis/Habituation.* The olfactory dis/habituation test was used to assess olfactory sensation as previously described[4]. Mice were placed in a cage. Cotton swabs were dipped in odorant and lowered into the cage for 60 seconds, followed by a 2-minute intertrial interval. Each odorant had 3 trials in a row to habituate the animal. Then a novel odorant was introduced and repeated for a total of 3 trials. The number of sniffs was recorded. The first odorant was always water, followed by isoamyl acetate or ethyl acetate in a counterbalanced fashion.

*Olfactory Memory*. The olfactory memory test was used to measure the memory retention of a familiar odor as previously described[4]. Stimuli were presented in the same setup as for olfactory dis/habituation. Animals were first exposed to ethyl vanillin for a period of 4 minutes to habituate them to the stimulus. An hour later, animals were exposed to ethyl vanillin again for a second trial of 4 minutes. If the animal remembers the odorant, they are expected to sniff the stimulus less during the second trial than the first.

*Whisker Brushing*. Whisker brushing was performed as previously described[5]. Briefly, animals were scruffed in one hand while the other brushed the distal end of the whiskers on each side of the face. Turning the face during or just after whisker brushing was considered indicative of a response.

*Footprint Test*. The footprint test was used to assess motor coordination and balance as previously described[6]. Animals were placed in a corridor (70 cm long) lined with paper. First, animals underwent a habituation phase (2, 10 minute sessions) to train them to run the corridor. A chunk of milk chocolate was placed at the end of the corridor. The end of the corridor was covered to make it dark. Animals were

scruffed and front paws were painted one color and hind paws were painted a different color. Animals were then placed at the beginning of the corridor and allowed to explore the corridor. If the animal reached the end of the corridor and the chocolate reward, they were placed back at the start with a fresh piece of chocolate at the end. In the test phase, the corridor was lined with fresh paper and a piece of chocolate. The animal's paws were painted and then the animal was placed at the start. As soon as the animal reached the end of the corridor or turned around in the corridor, the animal was removed and the trial was over. The test phase was repeated for a total of three trials or until a clear set of footprints with the animal moving in a straight path was obtained. Output measures were stride length (distance between footfalls with the same foot), overlap (distance between the center of the plantar of the fore and hind limb on the same stride), and base width (distance between the fore or hind feet on the same stride).

*AAV Generation and Delivery*. The AAV2/8 serotype was used because of its efficient gene delivery to the central nervous system of neonatal mice[7,8]. Cetalomegalovirus (CMV) was selected as the promoter because of its transduction efficiency in the central nervous system in neonatal rodents[9] and long-term expression[10]. A woodchuck posttrascriptional regulatory element (WPRE) was included to enhance gene expression[11]. The P2A peptide permitted separate expression of Cre recombinase and tdTomato[12]. TdTomato was used as the reporter because of its brightness and photostability[13].

Mice were initially assigned to a group based on visual estimation of sex on postnatal day 2. A few supplemental mice received viral infusions for each group due to anticipated exclusion of a subset of animals due to misplaced infusions. Postnatal day 2 mice were removed from the home cage and placed on a heating pad. Animals were cryoanesthetized on ice prior to surgery. The head was placed on a chin rest custom fitted into a stereotaxic device (Stoelting, Wood Dale, IL, USA). Virus was administered using a syringe pump (Harvard Apparatus, Holliston, MA, USA) holding a 10 µl syringe (#1701, Hamilton Company, Reno, NV, USA) attached to plastic tubing and a 33-gauge cannula (Plastics One, Roanoke, VA, USA) that was secured into the stereotaxic device. Coordinates were measured relative to lambda. Infusions were performed bilaterally at a rate of 0.1 µl/min for a total infusion of 0.25 µl per side.

The cannula was left in place for an additional minute for diffusion of virus prior to slowly drawing up the cannula. Animals were then removed from the stereotaxic device, and paws were tattooed for identification before returning to the heating pad. Once fully recovered from anesthesia, animals were rolled in soiled bedding from the home cage and returned to the home cage.

Following completion of behavioral testing, brains were extracted, fixed in 4% paraformaldehyde, and stored at -80°C. Brains were sectioned on a cryostat (Model 3050S; Leica Biosystems, Nussloch, Eisfeld, Germany). Sections were collected at 30 μm thickness onto Superfrost slides (Fisher) and mounted with Vectashield Hardset Antifade Mounting Medium with DAPI (Cat# H-1500; Vector Laboratories, Burlingame, CA, USA). Sections were then visualized under a fluorescent microscope (BX51; Olympus, Center Valley, PA, USA). Brains were assessed for infusion location by visualizing tdTomato. Mice were only included in analyses if the majority (>50%) of fluorescence was within the confines of the hippocampus.

*Dendritic Morphology*. Dendritic morphology of dentate granule neurons, CA1 pyramidal neurons, ACC layer II/III pyramidal neurons, and mediodorsal thalamus spiny stellate neuron dendrites were assessed in female *Btbd3* WT, HT, and KO mice (n = 6 WT, 5 HT and 5 KO mice, all female). Mice were transcardially perfused with 0.9% NaCl. Brains were then processed for Golgi-Cox staining and neurons were imaged and reconstructed as previously described[14]. Brains were extracted and immersed in Golgi-Cox solution, consisting of a 1:1 solution of 5% potassium dichromate and 5% mercuric chloride diluted 4:10 with potassium chromate for 14 days at room temperature[15]. Brains were then transferred to a 30% sucrose solution and stored at 4°C until shipped to Dr. Joao Bessa (University of Minho, Braga, Portugal) for processing. Brains were cut coronally at 200 μm section thickness on a vibratome. Sections were collected in 6% sucrose, blotted dry, and mounted onto gelatin-coated slides. Slides were then alkalinized in 18.7% ammonia. Next, slides were developed in Dektol (Kodak, Rochester, NY) and fixed in Kodak Rapid Fix. Slides were dehydrated in ethanol and cleared in xylene before coverslipping. Images were taken of each selected neuron at 600× magnification using a motorized microscope (Axioplan 2, Carl

Zeiss, LLC, USA) and a camera (DXC-390, Sony Corporation, Tokyo, Japan) and reconstructed using Neurolucida software (MBF Bioscience, Williston, VT) to obtain the complete dendritic tree. Three-dimensional analysis of the reconstructed neurons was performed using NeuroExplorer software (MBF Bioscience). Sholl analysis was performed on both apical and basal dendrites for pyramidal neurons. Spine density was assessed for all dendrites except on mediodorsal thalamus spiny stellate neurons due to insufficient resolution in the limited dendritic tree. Measurements were averaged across neurons within a subject, and this averaged measurement for each animal was used as the unit of analysis for statistical testing.

*Fluoxetine Metabolism*. Male *Btbd3* WT, HT, and KO mice were treated with fluoxetine in the drinking water at a concentration of 80 mg/L for a target dose of 10 mg/kg/day. After four weeks, animals were sacrificed and trunk blood was collected in tubes coated with EDTA. Samples were spun down and plasma layer pipetted off into new tubes. Plasma samples were sent to the Analytical Psychopharmacology Laboratories to measure fluoxetine and norfluoxetine levels using liquid chromatography with fluorescence detection (Nathan Kline Institute, Orangeburg, NY, USA)[16].

*Btbd3 qPCR*. *Btbd3*$^{flox}$ mice were infused with Cre recombinase or control virus (see main methods) into hippocampus at P2. Brains were extracted and snap frozen at eight weeks. Tissue punches were taken from hippocampus and stored at -80°C until RNA purification. Placement of viral infusion was verified during tissue punching by shining a UV light briefly over the tissue and visualizing fluorescence in hippocampus. RNA was isolated with the RNeasy Lipid Tissue Mini Kit (Qiagen, Germantown, MD) according to the manufacturer's instructions. qPCR was performed using the KAPA SYBR FAST One-Step assay (KAPA Biosystems, Wilmington, MA, USA). BTBD3 primers: (5' to 3'): CGTAAGAAGCCAGCCAACTC and CCCAACCACAAAATGTACGTC (Integrated DNA Technologies, Inc., Coralville, IA, USA). B-actin was used as the reference gene (Integrated DNA Technologies, Inc.).

*Western blot*. *Btbd3*$^{flox}$ mice were infused with Cre recombinase or control virus (see main methods) into hippocampus at P2. Brains were extracted and snap frozen at eight weeks. Tissue punches were taken from hippocampus and stored at -80°C until cell lysis. Placement of viral infusion was verified during tissue punching as above. Cells were lysed and protein quantified using the BCA assay. 20 μg protein was loaded into NUPAGE 10% Bis-Tris gels (Cat#: NP0301; ThermoFisher Scientific), and run at 200 V for 50 minutes. Protein was then transferred to 0.45 μm PVDF membrane (Cat#: IPFL00010; Millipore Sigma, Billerica, MA, USA) at 30 V for 50 minutes. The membrane was washed in TBST and incubated in blocking buffer for 1 hour at room temperature. The membrane was then incubated in 1:200 rabbit anti-BTBD3 (Cat#: HPA042048; Atlas Antibodies, Bromma, Sweden) or 1:10,000 rabbit anti-GAPDH (Cat# 2118; Cell Signaling Technology, Inc., Danvers, MA, USA) primary antibody overnight at 4°C. The membrane was washed in TBST then incubated in 1:1000 HRP-conjugated anti-rabbit secondary antibody (Cat#: 7074S; Cell Signaling Technology, Inc.) for one hour at room temperature. The membrane was washed in TBST then incubated in chemiluminescent substrate (Cat#: PI34080; Fisher Scientific) for 5 minutes at room temperature before visualizing on film (Cat#: E3018; Denville Scientific Inc., Holliston, MA, USA). Film was scanned in a transparency scanner. Bands were analyzed in ImageJ[17]. Background was removed using a rolling ball radius of 200 pixels. Standard densitometry analysis was performed to normalize BTBD3 bands to GAPDH bands within each lane.

## RESULTS

### Barbering

Mantel-Cox log rank tests revealed significant effects of fluoxetine treatment compared to vehicle pooled across genotypes (Figure 1c; week 14: $X^2_{(1, n = 185)} = 7.58$; $p<.01$). Onset of effects of fluoxetine were determined by testing for effects of treatment at the earlier weeks. Fluoxetine reduced onset of barbering behavior at weeks 4 ($X^2_{(1, n = 185)} = 6.05$; $p<.05$), 5 ($X^2_{(1, n = 185)} = 5.45$; $p<.05$), 6 ($X^2_{(1, n = 185)} = 6.47$; $p<.05$), 9 ($X^2_{(1, n = 185)} = 4.84$; $p<.05$), 10 ($X^2_{(1, n = 185)} = 6.33$; $p<.05$), 11 ($X^2_{(1, n = 185)} = 6.18$; $p<.05$), 12

($X^2_{(1, n = 185)}$ = 7.71; p<.01) and 13 ($X^2_{(1, n = 185)}$ = 8.60; p<.005) in addition to week 14. Weeks 2 ($X^2_{(1, n = 185)}$ = .003; p = .95), 3 ($X^2_{(1, n = 185)}$ = 1.48; p = .2245), 7 ($X^2_{(1, n = 185)}$ = 2.88; p = .09) and 8 ($X^2_{(1, n = 185)}$ = 3.46; p = .06) did not show significant effects of fluoxetine treatment.

Cognitive Phenotypes

Mice were assessed in the Go/No-Go paradigm to measure response inhibition (n = 12 WT, 11 HT, 16 KO mice, all male). No effect of genotype was identified for learning the task, as measured by a repeated measures ANOVA of d' (Supplementary Figure 1a; $F_{(2,36)}$ = 1.05; p = .36). Genotype also did not affect false alarm rate (Supplementary Figure 1b). However, a main effect of genotype was found for premature responding ($F_{(2,36)}$ = 3.45; p<.05); post hoc tests revealed that *Btbd3* HT and KO mice had lower instances of premature responding than WT mice (Supplementary Figure 1c).

In the PLT, genotype did not alter the percent win-stay responses on the target hole (Supplementary Figure 1d; $F_{(2,34)}$ = 2.93; p = .07) or percent lose-shift responses on the non-target (Supplementary Figure 1e).

Prepulse Inhibition

PPI testing was performed to measure sensorimotor gating (n = 14 female/15 male WT, 15 female/14 male HT, 14 female/14 male KO mice). Genotype did not affect PPI (Supplementary Figure 1f; $F_{(2,80)}$ = .93; p = .39) or startle amplitude (Supplementary Figure 1g) in repeated measures ANOVAs ($F_{(2,80)}$ = 1.12; p = .33).

Open Field.

In constitutive *Btbd3* WT, HT, and KO mice (n = 17 female/27 male WT, 34 female/27 male HT, and 11 female/20 male KO mice), there was no effect of genotype on spatial *d* (Supplementary Figure 1h). However, a genotype by bin interaction (F(16,1040) = 2.57; p<.0001) in a repeated measures ANOVA and post hoc tests revealed that *Btbd3* KO mice traveled a greater total distance than WT or HT

mice in bins 1-4 (Figure 2a). However, post hoc ANOVAs showed main effects of bin for each genotype for distance traveled. Furthermore, no difference in locomotor habituation was found between genotypes, assessed as the percent distance traveled in bin 9 relative to bin 1 ($F_{(2,133)} = 0.822$; $p = .44$).

In *Btbd3* whole hippocampal KD mice, a viral condition by bin interaction was identified in a repeated measures ANOVA for total distance traveled (Figure 4d; $F_{(8,560)} = 4.22$; $p<.0001$). Post hoc tests revealed higher activity levels in mice receiving AAV-Cre mice than AAV-tdTomato within each bin. Post hoc ANOVAs revealed a main effect of bin within each viral condition. Thus, consecutive bins were compared within each viral condition to examine habituation to novelty (Figure 4d). A significant difference was found between distance traveled in bins 1 and 2, and between bins 2 and 3, within control mice. However, there were no differences in distance traveled between any two consecutive bins within *Btbd3* KD mice. Next, habituation was compared between viral conditions. AAV-Cre mice habituated less than AAV-tdTomato mice, as indicated by a greater percent total distance traveled in bin 9 relative to bin 1 ($F_{(1,72)} = 19.64$; $p<.0001$; AAV-Cre: bin 9 mean = 49% of bin 1 activity, SEM = 9.1%; AAV-tdTomato: bin 9 mean = 13% of bin 1 activity, SEM = 1.9%).

For vertical rearing (Figure 4e), an interaction between bin and viral condition was identified ($F_{(8,560)} = 3.93$; $p<.0005$) in a repeated measures ANOVA. Post hoc ANOVAs revealed a main effect of bin within each viral condition. Neither viral condition showed differences between consecutive bins. For time spent rearing (Figure 4f), an interaction between viral condition and bin ($F_{(8,560)} = 3.49$; $p<.001$) was found in a repeated measures ANOVA. Post hoc ANOVAs identified a main effect of bin within each virus condition. Post hoc tests revealed no significant differences between consecutive bins within either the control or Cre virus conditions.

Sensory and Motor Tests

We next screened *Btbd3* KO mice for deficits in basic sensory and motor functioning, as *Btbd3* is robustly expressed in regions of high sensory acuity[18] and in the cerebellum[19]. Animals were assessed for olfactory sensitivity in the olfactory dis/habituation paradigm (n = 12/genotype/sex except for female

WT, n = 11 mice). A main effect of trial was identified (Supplementary Figure 2a; $F_{(2,130)} = 13.72$; $p<.0001$) in a repeated measures ANOVA, but no effects of genotype were found. Animals were also assessed for olfactory memory in the olfactory memory paradigm. A main effect of genotype was identified for number of sniffs ($F_{(2,65)} = 7.45$; $p<.005$). Post hoc tests revealed that *Btbd3* KO mice sniffed more than HT or WT mice (Supplementary Figure 2b). However, no genotype by trial interaction was identified ($F_{(2,65)} = 1.39$; $p = .25$). Whisker brushing was performed to assess whisker reflexes (n = 15/genotype/sex). No effect of genotype was found for left (Supplementary Figure 2c) or right (Supplementary Figure 2d) whisker responsiveness. The footprint test was performed to assess effects of *Btbd3* expression on motor coordination (n = 12/genotype/sex). There was no effect of genotype on forelimb stride length (Supplementary Figure 2e; $F_{(2,65)} = 2.76$; $p = .07$). No effect of genotype was identified for hind-limb stride length (Supplementary Figure 2f). No effects of genotype were identified on fore- (Supplementary Figure 2g) or hind-limb base width (Supplementary Figure 2h), or overlap (Supplementary Figure 2i).

Bodyweight

A main effect of genotype was identified for bodyweight (Supplementary Figure 2j; $F_{(2,66)} = 26.30$; $p<.0001$) in an ANOVA. Post hoc tests revealed that *Btbd3* KO mice weighed significantly less than *Btbd3* WT or HT mice (WT: mean = 25.7 g, SEM = 1.0; HT: mean = 24.4 g, SEM = 0.9; KO: mean = 20.8 g, SEM = 0.8).

Dendritic Morphology

We assessed dendritic morphology in adult *Btbd3* mice. No effect of genotype was found for dendritic branching or spine density in dentate gyrus granule or CA1 pyramidal neurons (Supplementary Figure 3a-d). In mediodorsal thalamus stellate neurons, genotype did not affect dendritic branching (Supplementary Figure 3e; $F_{(2,12)}=1.96$; $p=.18$). In layer II/III pyramidal neurons of the ACC, a main effect of genotype was identified for apical dendritic branching (Supplementary Figure 3f; $F_{(2,13)}=3.98$;

$p<.05$), but post hoc tests did not reveal significant contrasts. However, *Btbd3* KO mice had greater spine density in ACC than other genotypes (Supplementary Figure 3g-h; $F_{(2,13)}=4.29$; $p<.05$). Lastly, basal dendritic branching was unaltered in all regions assessed (data not shown).

Fluoxetine Metabolism

Plasma fluoxetine levels were measured to determine whether genotype-specific effects of fluoxetine on barbering behavior might have been due to differential fluoxetine metabolism (n = 4 WT, 5 HT, 6 KO mice, all male). No effects of genotype were identified on plasma fluoxetine ($F_{(2,12)} = .62$; $p = .55$; WT: mean = 384.0 ng/ml, SEM = 92.5; HT: mean = 298.6 ng/ml, SEM = 43.7; KO: mean = 315.2, SEM = 32.7) or norfluoxetine levels ($F_{(2,12)} = .21$; $p = .82$; WT: mean = 490.0 ng/ml, SEM = 80.5; HT: mean = 492.0 ng/ml, SEM = 15.9; KO: mean = 457.8 ng/ml, SEM = 33.4).

## SUPPLEMENTARY REFERENCES


1 Eagle DM, Bari A, Robbins TW. The neuropsychopharmacology of action inhibition: cross-species translation of the stop-signal and go/no-go tasks. *Psychopharmacology (Berl)* 2008; **199**: 439–456.

2 Milienne-Petiot M, Kesby JP, Graves M, van Enkhuizen J, Semenova S, Minassian A *et al.* The effects of reduced dopamine transporter function and chronic lithium on motivation, probabilistic learning, and neurochemistry in mice: Modeling bipolar mania. *Neuropharmacology* 2017; **113**, **Part A**: 260–270.

3 Shanahan NA, Velez LP, Masten VL, Dulawa SC. Essential role for orbitofrontal serotonin 1B receptors in obsessive-compulsive disorder-like behavior and serotonin reuptake inhibitor response in mice. *Biol Psychiatry* 2011; **70**: 1039–1048.

4 Zou J, Wang W, Pan Y-W, Lu S, Xia Z. Methods to measure olfactory behavior in mice. *Curr Protoc Toxicol* 2015; **63**: 11.18.1-21.

5 Crawley JN. *What's wrong with my mouse?: behavioral phenotyping of transgenic and knockout mice*. Wiley-Liss, 2000.

6 Brooks SP, Trueman RC, Dunnett SB. Assessment of Motor Coordination and Balance in Mice Using the Rotarod, Elevated Bridge, and Footprint Tests. *Curr Protoc Mouse Biol* 2012; **2**: 37–53.

7 Broekman MLD, Comer LA, Hyman BT, Sena-Esteves M. Adeno-associated virus vectors serotyped with AAV8 capsid are more efficient than AAV-1 or -2 serotypes for widespread gene delivery to the neonatal mouse brain. *Neuroscience* 2006; **138**: 501–510.

8 Chakrabarty P, Rosario A, Cruz P, Siemienski Z, Ceballos-Diaz C, Crosby K *et al.* Capsid serotype and timing of injection determines AAV transduction in the neonatal mice brain. *PloS One* 2013; **8**: e67680.

9 McCown TJ, Xiao X, Li J, Breese GR, Jude Samulski R. Differential and persistent expression patterns of CNS gene transfer by an adeno-associated virus (AAV) vector. *Brain Res* 1996; **713**: 99–107.

10 Tenenbaum L, Jurysta F, Stathopoulos A, Puschban Z, Melas C, Hermens WT *et al.* Tropism of AAV-2 vectors for neurons of the globus pallidus. *Neuroreport* 2000; **11**: 2277–2283.

11 Wang L, Wang Z, Zhang F, Zhu R, Bi J, Wu J *et al.* Enhancing Transgene Expression from Recombinant AAV8 Vectors in Different Tissues Using Woodchuck Hepatitis Virus Post-Transcriptional Regulatory Element. *Int J Med Sci* 2016; **13**: 286–291.

12 Kim JH, Lee S-R, Li L-H, Park H-J, Park J-H, Lee KY *et al.* High cleavage efficiency of a 2A peptide derived from porcine teschovirus-1 in human cell lines, zebrafish and mice. *PloS One* 2011; **6**: e18556.

13 Shaner NC, Steinbach PA, Tsien RY. A guide to choosing fluorescent proteins. *Nat Methods* 2005; **2**: 905–909.

14 Cerqueira JJ, Taipa R, Uylings HBM, Almeida OFX, Sousa N. Specific Configuration of Dendritic Degeneration in Pyramidal Neurons of the Medial Prefrontal Cortex Induced by Differing Corticosteroid Regimens. *Cereb Cortex* 2007; **17**: 1998–2006.

15 Glaser EM, Van der Loos H. Analysis of thick brain sections by obverse-reverse computer microscopy: application of a new, high clarity Golgi-Nissl stain. *J Neurosci Methods* 1981; **4**: 117–125.

16 Suckow RF, Zhang MF, Cooper TB. Sensitive and selective liquid-chromatographic assay of fluoxetine and norfluoxetine in plasma with fluorescence detection after precolumn derivatization. *Clin Chem* 1992; **38**: 1756–1761.

17 Schneider CA, Rasband WS, Eliceiri KW. NIH Image to ImageJ: 25 years of image analysis. *Nat Methods* 2012; **9**: 671–675.

18 Matsui A, Tran M, Yoshida AC, Kikuchi SS, U M, Ogawa M *et al.* BTBD3 controls dendrite orientation toward active axons in mammalian neocortex. *Science* 2013; **342**: 1114–1118.

19 Lein ES, Hawrylycz MJ, Ao N, Ayres M, Bensinger A, Bernard A *et al.* Genome-wide atlas of gene expression in the adult mouse brain. *Nature* 2007; **445**: 168–176.
