# Supplementary material for: Btbd3 expression regulates compulsive-like and exploratory behaviors in mice
Source: Transl Psychiatry. 2019 Sep 9;9:222. doi: 10.1038/s41398-019-0558-7 (PMC6733800; doi:10.1038/s41398-019-0558-7)
Supplement: Supplementary file 2 — Supplementary Figures. [file 41398_2019_558_MOESM2_ESM.pdf]

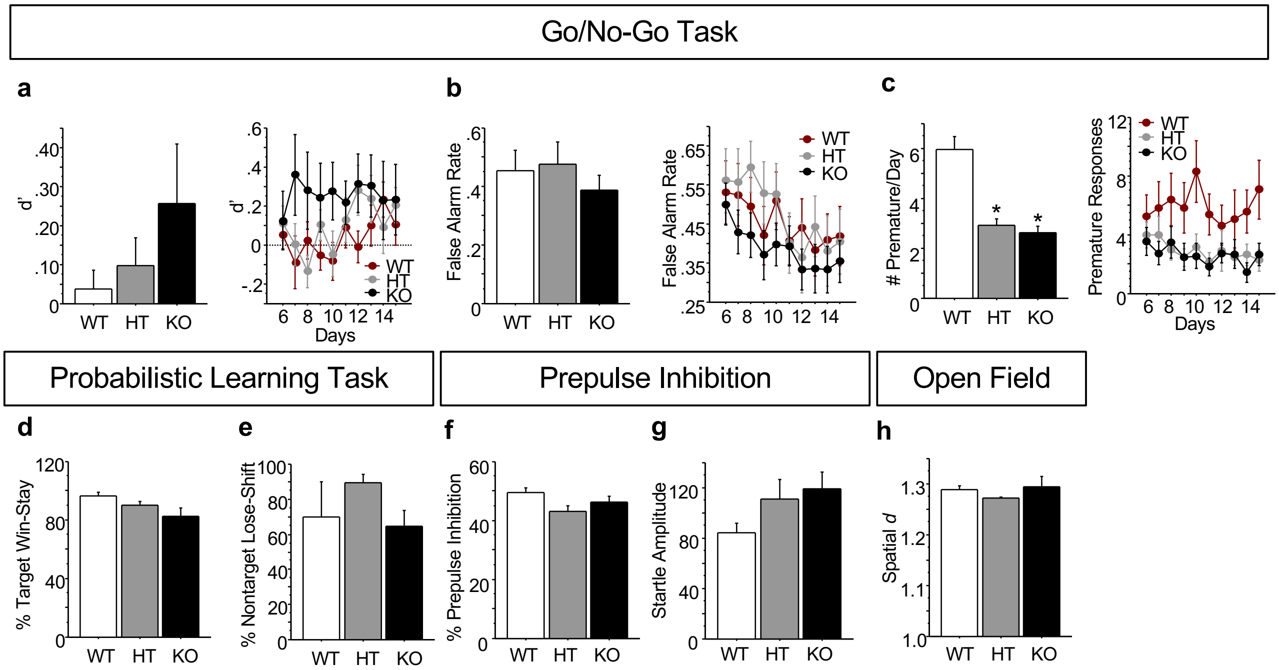

Supplementary Figure 1. Effects of *Btbd3* knockout on additional behavioral measures. In the Go/No-Go paradigm (n = 12 WT, 11 HT, 16 KO mice, all male), there was no difference between genotypes for discrimination index *d'* overall (a, left) or across days (a, right). *Btbd3* genotype did not affect false alarm rate overall (b, left) or across days (b, right), but did affect premature responses, with an overall reduction in *Btbd3* HT and KO mice (c, left) that did not differ across days (c, right). *Btbd3* genotype did not affect win-stay responses on the target port (d) or lose-shift responses on the non-target port (e) in the PLT (n = 13 WT, 16 HT, 17 KO mice, all male). No differences in prepulse inhibition (f) or acoustic startle amplitude (g) were identified between genotypes (n = 14 female/15 male WT, 15 female/14 male HT, 14 female/14 male KO mice). *Btbd3* genotype did not affect spatial *d* in the open field (n = 17 female/27 male WT, 34 female/27 male HT, and 11 female/20 male KO mice) . Results are expressed as mean values ± SEM. *p<.05 vs WT group as determined by ANOVA and post hoc tests. WT: wild-type HT: heterozygous KO: knockout PLT: probabilistic learning task.

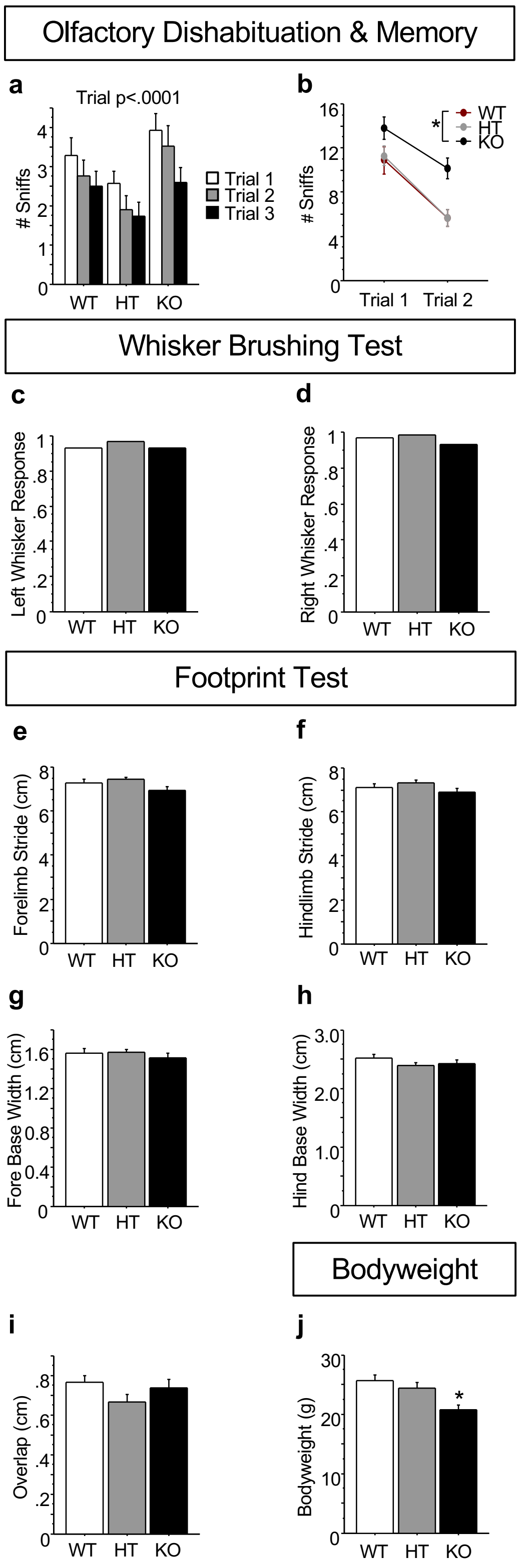

Supplementary Figure 2. *Btbd3* knockout does not have robust effects on sensory or motor capabilities (n = 12/genotype/sex except for female WT, n = 11 mice). In the olfactory dis/habituation test (a), no effect of *Btbd3* genotype was identified for habituation to repetition of an odor or dishabituation when a new odor was introduced. In the olfactory memory test (b), no effect of *Btbd3* genotype on olfactory memory was found, but an overall increase in instances of sniffing was seen in *Btbd3* KO mice. No effect of genotype on left (c) or right (d) whisker response was found (n = 15/genotype/sex). No effect of *Btbd3* genotype was identified in the footprint test (n = 12/genotype/sex) for any measure (e-i). Young adult *Btbd3* KO mice had significantly reduced bodyweight relative to WT mice (j). Results are expressed as mean values ± SEM, except for panels depicting categorical data, which does not have error. *p<.05 vs WT group as determined by ANOVA and post hoc tests. WT: wild-type HT: heterozygous KO: knockout.

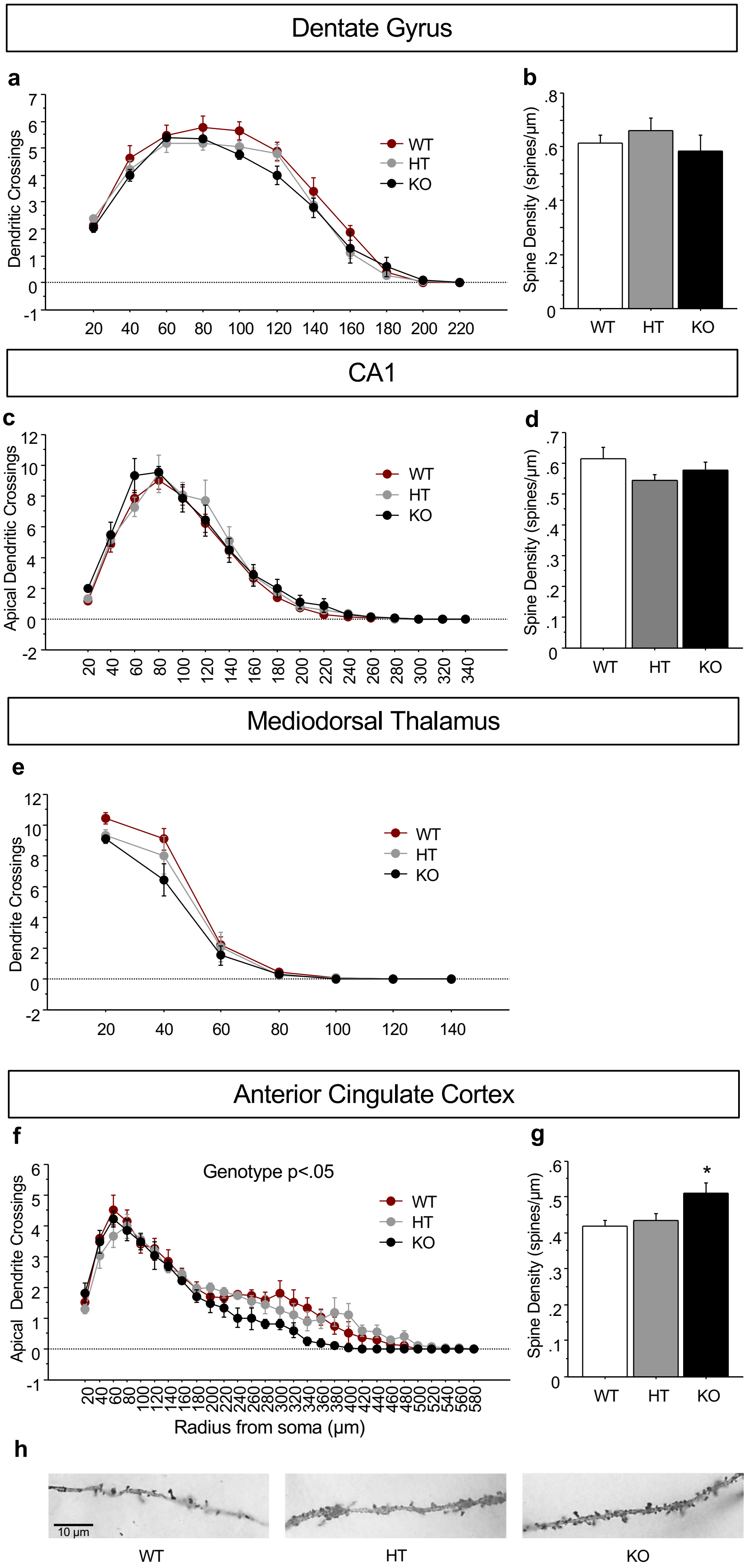

Supplementary Figure 3. *Btbd3* knockout does not cause major alterations in limbic CSTC circuit dendritic morphology. Apical dendritic branching was unaffected by *Btbd3* genotype in granule neurons of the dentate gyrus (n = 6 WT, 5 HT, 5 KO mice, all female) (a), CA1 pyramidal neurons (c), and spiny stellate neurons in mediodorsal thalamus (e). Spine density was also unaffected by *Btbd3* genotype in the hippocampus, in both the dentate gyrus (b) and CA1 (d). Layer II/III pyramidal neurons in ACC showed a mild effect of *Btbd3* genotype on apical dendritic branching, but with no specific differences between groups (f). Spine density was elevated in *Btbd3* KO ACC layer II/III pyramidal neurons relative to WT mice (g). Representative dendritic spine images from ACC layer II/III pyramidal neurons (h). Results are expressed as mean values ± SEM. *p<.05 vs WT group determined by ANOVA and post hoc tests. WT: wild-type HT: heterozygous KO: knockout ACC: anterior cingulate cortex.
